# Supplementary material for: Reversible assembly and disassembly of V-ATPase during the lysosome regeneration cycle
Source: Mol Biol Cell. 2024 Apr 2;35(5):ar63. doi: 10.1091/mbc.E23-08-0322 (PMC11151095; doi:10.1091/mbc.E23-08-0322)
Supplement: Supplementary file 6 [file reviewer_reportE23-08-0322.pdf]

Reversible assembly and disassembly of V-ATPase during the lysosome regeneration cycle

Ioana Sava, Luther Davis, Sally Gray, Nicholas Bright, and J. Paul Luzio

Corresponding author(s): J. Paul Luzio, University of Cambridge, UK

Review Timeline:

Submission Date:	2023-08-18
Editorial Decision:	2023-09-29
Revision Received:	2024-02-22
Accepted:	2024-02-26

Editor-in-Chief: Matthew Welch

Transaction Report:

(Note: With the exception of the correction of typographical or spelling errors that could be a source of ambiguity, letters and reports are not edited. The original formatting of letters and referee reports may not be reflected in this compilation.)

RE: Manuscript #E23-08-0322
TITLE: Reversible assembly and disassembly of V-ATPase during the lysosome regeneration cycle

Dear Paul,

Thank you for submitting your manuscript, "Reversible assembly and disassembly of V-ATPase during the lysosome regeneration cycle", to be considered for publication in the Special Issue on The Cell Biology of the Lysosome in Molecular Biology of the Cell. I apologize for the unusually long time in review. I asked two respected colleagues to review the manuscript, and their verbatim comments are appended below. As you'll see, both reviewers are very enthusiastic (as am I!) about the manuscript and feel that it has the potential to be an important contribution for the field. However, both reviewers also raise a number of important concerns that will need to be addressed before recommending publication. In particular, both authors seek a bit more information regarding the mechanism by which the vATPase V1 subcomplex is recruited to sucrosomes. Indeed, both suggest the same experiment to assess the contribution of altered intralumenal pH to this process. I agree that further insight into recruitment would be an important addition to this paper, and that the suggested experiment should be included. Reviewer 1 suggests some additional experiments to test the role of activated RAB7 in this process, and I suspect that these will not be difficult for you to do. Having said that, however, if these experiments prove negative, my sense is that the reviewers do not seek a wild goose chase after the mechanism. Thus, if they fail to elucidate a requirement for deacidification or Rab7 in V1 recruitment, I would not insist on additional approaches to probe the mechanism. Both reviewers very clearly point out several additional concerns that I will not reiterate here and that should be easy to address in the text or with additional data that you likely have on hand.

While I cannot accept the manuscript in its current form, I would be very pleased to consider a revised manuscript that addresses the reviewers' concerns. When you resubmit, please provide a point-by-point response to each of the reviewers' points and, if possible, please indicate (e.g. by a different colored font) where changes to the text have been made. Given the clarity of the reviewers' concerns, it is likely that I will be able to make a decision on the revised manuscript without additional re-review.

Sincerely,

Mickey

Michael Marks, PhD
Monitoring Editor
Molecular Biology of the Cell

Dear Prof. Luzio,

The review of your manuscript, referenced above, is now complete. The Monitoring Editor has decided that your manuscript is not acceptable for publication at this time, but may be deemed acceptable after specific revisions are made, as described in the Monitoring Editor's decision letter above and the reviewer comments below.

A reminder: Please do not contact the Monitoring Editor directly regarding your manuscript. If you have any questions regarding the review process or the decision, please contact the MBoC Editorial Office (mboc@ascb.org).

When submitting your revision include a rebuttal letter that details, point-by-point, how the Monitoring Editor's and reviewers' comments have been addressed. (The file type for this letter must be "rebuttal letter"; do not include your response to the Monitoring Editor and reviewers in a "cover letter.") Please bear in mind that your rebuttal letter will be published with your paper if it is accepted, unless you have opted out of publishing the review history.

Authors are allowed 90 days to submit a revision. If this time period is inadequate, please contact us at mboc@ascb.org.

Revised manuscripts are assigned to the original Monitoring Editor whenever possible. However, special circumstances may preclude this. Also, revised manuscripts are often sent out for re-review, usually to the original reviewers when possible. The Monitoring Editor may solicit additional reviews if it is deemed necessary to render a completely informed decision.

In preparing your revised manuscript, please follow the instruction in the Information for Authors (www.molbiolcell.org/info-for-authors). In particular, to prepare for the possible acceptance of your revised manuscript, submit final, publication-quality figures with your revision as described.

If your manuscript contains a Significance Statement, please make sure that it consists of three separate bullet points totaling a maximum of 100 words and addressing each of the following points (see https://www.molbiolcell.org/curation-tools):
First bullet: What is the background context? What gap in knowledge does this study addres?
Second bullet: What are the key findings? What is unique or new about the approach?
Third bullet: Why is this paper significant? How might it influence future research?

To submit the rebuttal letter, revised manuscript, and figures, use this link: Link Not Available

MBoC offers the option to publish your work with immediate open access. Open access can increase the discoverability and usability of your research. If you would like to publish your paper with immediate open access but did not select this option during initial submission, please contact the editorial office at mbc@ascb.org.

Thank you for submitting your manuscript to MBoC. We look forward to receiving your revised paper.

Sincerely,

Eric Baker
Journal Production Manager
MBoC Editorial Office
mbc@ascb.org

Reviewer #1 (Remarks to the Author):

The manuscript by Sava et al. analyzes the assembly and disassembly of the V-ATPase in lysosomes and in sucrosomes, lysosomes enlarged by overnight incubation with sucrose, which is not metabolizable and not transportable and accumulates imposing an osmotic strain that arrests the lysosome regeneration cycle. The main finding is that the association of the V1 subunit of the V-ATPase with the V0 subunit increases markedly in sucrosomes, compared to normal lysosomes, and recovers following addition of invertase that degrades sucrose into transportable monomers.

The work is thorough, carefully performed and quantified and presented very clearly. The findings are compelling but there are some questions that need to be addressed.

Specific comments
1) It is ultimately unclear what is the determinant of V1 recruitment to the sucrosomes. The increased assembly correlates with increased Rab7, but a causal relationship was not established. Does expression of constitutively active Rab7, which I believe increases moderately the size of lysosomes, result in increased assembly? Does the dominant-negative allele have the opposite effect; indeed do cells expressing the T22N mutant lack acidic lysosomes? How do earlier compartments that ostensibly lack Rab7 acidify?
2) The authors find that only ≈50% of the LAMP1 colocalizes with Rab7. Is this finding typical? It was my impression that the colocalization of the two markers is almost complete in other reports. Are the findings reported here affected by the heterologous overexpression of LAMP1? Have the authors confirmed the extent of colocalization with detection of endogenous LAMP?
3) How is the excess Rab7 recruitment to sucrosomes explained? The authors may have explored (and published?) this in the past, but unfamiliar readers would benefit from a more detailed explanation of the underlying processes.
4) The authors assume that the excess V1 recruited to sucrosomes is bound by the V0. This is a reasonable assumption, especially considering the somewhat more acidic pH of the sucrosomes. But other variables can account for the acidification (e.g. reduced leak). Can the authors rule out that V1 components are recruited by other means to sucrosomes (e..g. non-specifically). This possibility is difficult to test experimentally but should perhaps be acknowledged in the revised text.
5) In the kiss and fusion experiments, could the V1 subunit that the authors visualized be acquired by fusion of holoenzyme (i.e. along with the V0 subunit) rather than assembly with pre-existing V0?
6) Have the authors tested if the V-ATPase catalytic function is required for the continuous disassociation of V1 from V0 and for the additional dissociation upon invertase treatment? Does inhibition with bafilomycin or concanamycin affect the recovery in FRAP experiments?

Minor comments
1) What is the gold labeling in the CLEM experiments? It is not clearly stated either in the text or figure legend.
2) Do the authors believe that the cathepsins are recycled in inactive form in the retrieval pathway or are they segregated from the recycling membranes in some way? If so how? Does the BMV109 reagent attach only to the active cathepsins and are these present but inactive in the recycling compartment? Have the authors immunostained for total cathepsins in the BMV109-negative compartment?
3) If not dynasore-sensitive, can the authors suggest alternative mechanisms for tubulation and sorting of the material removed from the sucrosomes?

Reviewer #2 (Remarks to the Author):

In the current manuscript, Sava et al analyze the reversible assembly of the vacuolar H+ ATPase (V-ATPase) under conditions that induce endolysosome fusion and subsequent lysosome reformation. They propose a model in which endolysosomal fusion and the resulting content mixing trigger an increase in assembly of the V-ATPase soluble V1 sector with the membrane-embedded V0 sector, thereby leading to more proton pumping, acidification and activation of hydrolytic enzymes (i.e. cathepsins). Following lysosomal reformation via tubulation, the assembly status of V-ATPase returns to baseline.
The manuscript is generally well executed and provides a valuable analysis of V-ATPase dynamics and its relationship to endolysosomal fusion and trafficking in living cells.
A few issues are noted that should be addressed.

1- The V1G1-EGFP and V0a3-EGFP reporters should be validated biochemically for correct incorporation in their respective subdomains. Do they pull down other components of the V1 and V0 sector, respectively, in co-immunoprecipitation assays?
2- The authors state that autophagy is not triggered by sucrose feeding of NRK cells, but provide no evidence. Given this is likely a major stress, its effect on autophagy should be analyzed. Better still, they could repeat their analysis in NRK cells that are incapable of triggering autophagy due to deletion of an essential regulator (i.e. ATG5).
3- Ultimately, the mechanism that couples endolysosomal fusion and content mixing to changes in V-ATPase assembly remains unexplained. One possibility is that the loss of internal acidity due to increased volume (and thus proton dilution) upon fusion may be the stimulus. The authors could test that by measuring V1G1 membrane association and FRAP behavior when cells are treated with agents that disrupt lysosomal acidification, either by directly inhibiting the V-ATPase (i.e. bafilomycin A) or indirectly (i.e. chloroquine).

University of Cambridge
Cambridge Institute for Medical Research,
The Keith Peters Building,
Cambridge Biomedical Campus,
Hills Road, Cambridge CB2 0XY, UK

21st February 2024

Dear Mickey,

Re: Manuscript #E23-08-0322

TITLE: Reversible assembly and disassembly of V-ATPase during the lysosome regeneration cycle

Thank you for your email of 29th September and the reviews of our manuscript. It is reassuring to know that both the reviewers and you are enthusiastic about it. We have carried out some further experiments to address the points raised by the reviewers. These include experiments to address the two matters mentioned in particular in your own letter, i.e. the contribution of altered intraluminal pH to V-ATPase assembly/disassembly and the role of activated RAB7a. In revised supplemental figures S3 and S5 we have added panels (new panels S3F-H and S5I) showing the results of these experiments and amended the text in both Results and Discussion accordingly. Although the additional experiments have proved informative, I think we are still some distance from showing the molecular mechanism linking RAB7a to V-ATPase assembly/disassembly, which we will continue to work on in the future. I have provided below a point-by-point response to the reviewers' comments. Where the text of the revised manuscript has been altered this is indicated by using a different coloured font as you have suggested. I have added the revised supplemental figure legends to the end of this letter so that you can see from the colored text the changes to the legends for figures S3 and S5 and these have also been corrected without color in the new pdf of the revised Supplemental Material.

I look forward to hearing your decision on the revised manuscript.

Sincerely,

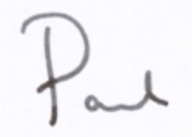

J.Paul Luzio

Response to Reviewer #1 Specific Comments

1) *It is ultimately unclear what is the determinant of V1 recruitment to the sucrosomes. The increased assembly correlates with increased Rab7, but a causal relationship was not established. Does expression of constitutively active Rab7, which I believe increases moderately the size of lysosomes, result in increased assembly? Does the dominant-negative allele have the opposite effect; indeed do cells expressing the T22N mutant lack acidic lysosomes? How do earlier compartments that ostensibly lack Rab7 acidify?*

To address these questions, we transiently expressed 2HA tagged RAB7aWT, RAB7aQ67L and RAB7aT22N into NRK cells, which are either stably expressing LAMP1-mCherry and V1G1-EGFP or pHLARE. We have added a paragraph on page 8 of the revised manuscript to describe the results of these experiments along with 3 new panels, F-H, in Fig S3 showing

the data and have also modified our Discussion accordingly on pages 15/16 of the revised manuscript. We saw an increased Manders' colocalization coefficient for V1G1-EGFP vs LAMP1-mCherry with the Q67L (constitutively active) mutant and reduced colocalization with the T22N (dominant negative) mutant consistent with active RAB7a being required for increased V-ATPase assembly. Measurements of pH using the pHLARE construct showed that expression of the Q67L mutant made the pHLARE-labelled organelles more acidic and T22N made them less acidic than in control cells. Cells transiently transfected with all three RAB7a constructs still made sucrosomes when incubated with 30mM sucrose and these sucrosomes resolved after uptake of invertase. In the cells expressing the RAB7a mutants, we were unable to obtain reliably reproducible data to show changes and/or differences in changes of colocalization of V1G1-EGFP vs LAMP1-mCherry after sucrosome formation, and subsequent uptake of invertase. We believe this was in part because the colocalization was already significantly different in the control cells expressing the mutants and because of a greater spread of data from individual cells as a result of using a transient transfection protocol for the RAB7a mutants. In the pHLARE-expressing cells we did see that whereas the pH of the swollen sucrosomes became more acidic than the pH of late/endosomes in untreated cells and returned to untreated values after subsequent incubation with invertase, there was no significant change in the more acidic pH seen when Q67L RAB7a was expressed or the less acidic pH seen in the presence of the T22N mutant. Our pH data from 3 separate experiments (10 cells each) are summarised in the following table showing mean pH±SEM of organelles in pHLARE-expressing cells:

	Control	+ RAB7aWT	+RAB7aQ67L	+RAB7aT22N
Untreated	5.14±0.13	4.92±0.18	4.55±0.14	5.91±0.16
Sucrose(30mM) 24h	4.76±0.17	4.46±0.16	4.51±0.17	5.67±0.15
Sucrose(30mM) 24h and then 0.5 mg/mL invertase 8h	5.13±0.14	4.86±0.18	4.80±0.18	5.79±0.18

We did not investigate the acidification of earlier endocytic compartments that lack RAB7a. These compartments are much less acidic than RAB7a-positive late compartments. In addition, it has been reported that the V-ATPase in early compartments has a different composition from that of later compartments with respect to the isoforms of the Voa subunit[1]. Whether this has any bearing on the mechanism of V-ATPase assembly, V-ATPase regulation and/or regulation of lumenal pH we do not know.

2) *The authors find that only ≈50% of the LAMP1 colocalizes with Rab7. Is this finding typical? It was my impression that the colocalization of the two markers is almost complete in other reports. Are the findings reported here affected by the heterologous overexpression of LAMP1? Have the authors confirmed the extent of colocalization with detection of endogenous LAMP?*

When localization of RAB7A was first studied, it was shown by immunoEM in BHK cells that "rab7 was only found associated with the CI-MPR-positive late endosomes, while the CI-MPR-negative lysosomes,…. were invariably devoid of labelling"[2]. Subsequently, confocal microscopy of HeLa cells showed a high degree of colocalization of EGFP-tagged RAB7a with LAMP1[3], although more recently it was found that peripheral lysosomes have a lower density of EGFP-tagged RAB7a than central lysosomes[4]. Studies reporting confocal

microscopy-based quantitative estimates of RAB7A colocalization with LAMP1, in more than one cell type, have shown that the extent of colocalization may be very cell type dependent. In one study 70% of endo-lysosomal vesicles were shown to be positive for both RAB7a and LAMP1 in BS-C-1 cells and 91% in HeLa cells[5]. In another study colocalization of RAB7a and LAMP1 in C2C12 cells was 74-86%, but only 26-37% in A549 cells[6]. The simplest interpretation of our Manders' colocalization coefficients would, as the reviewer indicates, suggest that we have ~50% colocalization of EFGP-tagged RAB7a and mCherry-tagged LAMP1 in our stably transfected NRK cells, although one has to be cautious about over-interpreting Manders' coefficients as % colocalization, not least because of the problems of spatial variation of background and noise, which affect thresholding and can lead to under- or over-estimates of colocalization[7]. We do not believe our findings were affected by the heterologous overexpression of LAMP1-mCherry. This is because in fixed cells stably expressing only RAB7a-EGFP, we see similar colocalization of RAB7a-EGFP with both endogenous LAMP1 and LAMP2 to the colocalization observed with LAMP1-mCherry in the LAMP1-mCherry/V1G1-EGFP cells. Data from a single set of experiments is shown below in Fig A:

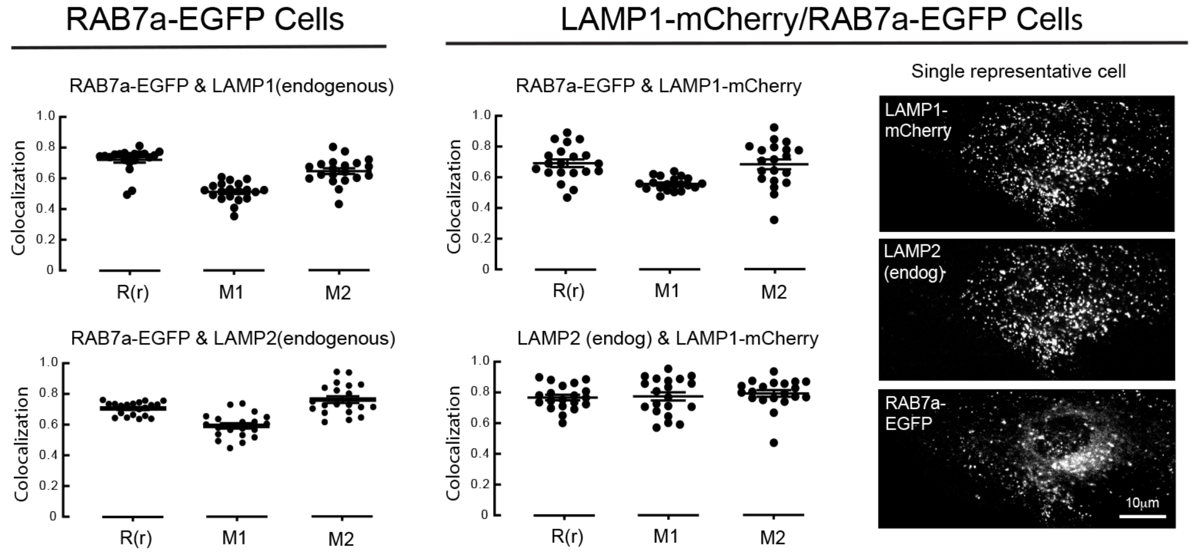

Fig A. Colocalization of RAB7a-EGFP with LAMPs. Left hand panels show Pearson's (R(r)) and Manders' colocalization coefficients (M1 and M2) for RAB7a-EGFP with endogenous LAMP1 and LAMP2 in NRK cells stably expressing RAB7a-EGFP at 1.3x endogenous RAB7a. Right hand panels show colocalization coefficients for RAB7a-EGFP and endogenous LAMP2 with LAMP1-mCherry in mixed stable LAMP1-mCherry/RAB7a-EGFP cells (described in Supplemental Table 1 of manuscript) together with representative confocal fluorescence images. All cells were fixed with 4% paraformaldehyde and permeabilised with 0.05% saponin. Primary antibodies used for indirect immunofluorescence localization were monoclonal mouse anti-GFP (ma5-15256, Invitrogen/Thermofisher Scientific) for RAB7a-EGFP, rabbit polyclonal anti-LAMP1 (ab21470, Abcam) and rabbit polyclonal anti-rat LAMP2 (lgp110)[8]. Intrinsic fluorescence was used to localize LAMP1-mCherry. 20 cells were analyzed in each case for colocalization coefficients and mean±SEM shown.

3) *How is the excess Rab7 recruitment to sucrosomes explained? The authors may have explored (and published?) this in the past, but unfamiliar readers would benefit from a more detailed explanation of the underlying processes.*

What we observed was an increased colocalization of RAB7a-EGFP with LAMP1-mCherry when sucrosomes (swollen endolysosomes) formed. The simplest explanation is that this is simply a reflection of the fusion of RAB7a-positive late endosomes with RAB7a-

negative(depleted)/LAMP1-positive lysosomes and 'subsequent' fusions of additional Rab7+ve endosomes into the swollen sucrosomes, from which lysosome reformation is blocked. It is possible that this is too simplistic an explanation – for example we have not formally assessed whether there is additional recruitment from any cytosolic pool of RAB7a-EGFP, but we have not explored this further. We had already included a sentence (page 16 of the revised manuscript) to explain the recruitment of RAB7a to sucrosomes and this stated, "Our colocalization data do not distinguish between either the increased colocalization of RAB7a with LAMP1 after formation of sucrosomes being simply a reflection of the recruitment of peripheral RAB7a-depleted/LAMP1-enriched lysosomes into the swollen sucrosomes as a result of fusion events with RAB7a-positive late endosomes or the direct recruitment from a cytosolic pool onto the sucrosomes, although we did not investigate this further." We have slightly amended the text in the revised manuscript to make it clearer that we favour the first explanation.

4) *The authors assume that the excess V1 recruited to sucrosomes is bound by the V0. This is a reasonable assumption, especially considering the somewhat more acidic pH of the sucrosomes. But other variables can account for the acidification (e.g. reduced leak). Can the authors rule out that V1 components are recruited by other means to sucrosomes (e..g. non-specifically). This possibility is difficult to test experimentally but should perhaps be acknowledged in the revised text.*
On page 10 of our revised manuscript, we have added a sentence to explain that our experiments have not formally excluded the recruitment of V1 to sucrosomes by means other than association with Vo.

5) *In the kiss and fusion experiments, could the V1 subunit that the authors visualized be acquired by fusion of holoenzyme (i.e. along with the V0 subunit) rather than assembly with pre-existing V0?*
We think that acquisition of the V1subunit by fusion of holoenzyme is extremely unlikely. In our sucrosome experiments the colocalization of Voa3-EGFP with LAMP1-mCherry remained constant during sucrosome formation and subsequent invertase uptake (lysosome reformation), in contrast to the changes in colocalization of V1G1-EGFP with LAMP1-mCherry. The evidence that we were recruiting/losing V1 subunits during sucrosome formation/lysosome reformation was backed up by our immunoblotting data from magnetically isolated organelles. All our evidence is consistent with recruitment of V1 subunits from a cytosolic pool during sucrosome formation and it is known that in mammalian cells the cytosolic pool of V1 subunits is large[10]. In our experiments with transfected NRK cells, we found, by immunoblotting 100,0000g supernatants and membrane pellets after cell homogenisation and centrifugation, that whereas Voa3-EGFP was in the membrane pellet and undetectable in the supernatant, 85.2±5.6% (6 separate experiments) of V1G1-EGFP was in the supernatant i.e. was cytosolic. Although we do not have data for the cytosol:membrane distribution of endogenous subunits of the V-ATPase in NRK cells, data are available for HeLa cells from the HeLa spatial proteome, http://mapofthecell.biochem.mpg.de. These data show the % of each endogenous subunit in the cytosol as being V1A, 83%; V1B2, 77%; V1C1, 77%; V1D, 78%; V1E1, 69%; V1F, 66%; V1G1, 76%; V1H, 85%; Voa1, 6% ; Voa3, 17%; Vod1, 11%; Voa2/Voa4/Voc, 0%. Thus, our figure for the cytosolic pool of V1-EGFP in the transfected NRK cells is within the range found for endogenous V1 subunits in HeLa cells.

6) *Have the authors tested if the V-ATPase catalytic function is required for the continuous disassociation of V1 from V0 and for the additional dissociation upon invertase treatment? Does inhibition with bafilomycin or concanamycin affect the recovery in FRAP experiments?*
We have repeated the FRAP experiment on V1G1-EGFP associated with the sucrosome membrane in the presence of 100nM bafilomycin A to inhibit the V-ATPase. The fluorescence recovery of V1G1-EGFP was not prevented, but slightly enhanced and this is now shown in a new supplemental figure panel (Fig S5I). In a single experiment shown below (Fig B), we saw no obvious effect of 100nM bafilomycin A on either the increase in colocalization of V1G1-EGFP with LAMP1-mCherry during sucrosome formation, nor the decrease in colocalization after delivery of invertase to sucrosomes. We have described/commented on these findings in the revised manuscript by additions to the text on pages 11/12 and 14/15 of the revised manuscript.

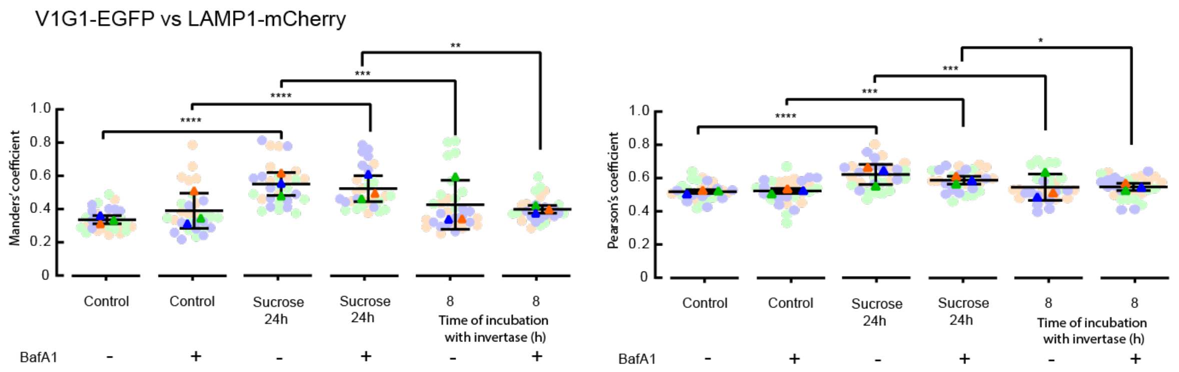

Fig B. Colocalization of V1G1-EGFP-EGFP with LAMP1-mCherry in the presence of Bafilomycin A1. Manders' (left panel) and Pearson's (right panel) colocalization coefficients for V1G1-EGFP vs LAMP1-mCherry in mixed stable NRK cells expressing the two constructs,which were either untreated, incubated with 30mM sucrose for 24h or incubated with sucrose followed by 8h incubation with 0.5 mg/mL invertase, each condition in the absence (-) or presence (+) of 100nM Bafilomycin A1. Bafilomycin A1 was present for 8h in otherwise untreated cells, for the final 8h of incubation with 30mM sucrose and throughout the 8h incubation with invertase. Mean±SEM of 3 experiments (minimum 10 cells per experiment) shown. *, $p<0.05$, **, $p<0.01$, ***, $p<0.005$; ****, $p<0.001$.

Minor comments

1) *What is the gold labeling in the CLEM experiments? It is not clearly stated either in the text or figure legend.*
We have amended the figure legend in Figure 2E to make it clearer that the gold particles identifying V1G1-EGFP were as a result of labelling with rabbit anti-GFP and protein A- gold (PAG), 10nm.

2) *Do the authors believe that the cathepsins are recycled in inactive form in the retrieval pathway or are they segregated from the recycling membranes in some way? If so how? Does the BMV109 reagent attach only to the active cathepsins and are these present but inactive in the recycling compartment? Have the authors immunostained for total cathepsins in the BMV109-negative compartment?*
We believe that inactive cathepsins in neutral terminal lysosomes become active in the acidic environment generated when they fuse with late endosomes to form endolysosomes or the acidic environment existing when they fuse with endolysosomes. The data in the present manuscript and our previous publication[9] are consistent with there being a

population of neutral terminal lysosomes that contain cathepsins, which are not active. In our previous study[9], we showed that the lack of hydrolysis of Magic Red cathepsin substrates in cathepsin-inactive terminal lysosomes was not due to a problem of substrate access or the absence of enzyme in these compartments, since incubation of the cells with a Magic Red cathepsin substrate in a pH 5 buffer in the presence of nigericin and monensin to equilibrate pH across compartments allowed us to capture images of the appearance of cresyl violet fluorescence in all of a cell's fluorescent dextran-positive terminal endocytic compartments before it dissipated across the organelles' limiting membranes within 5 min. Unfortunately, for that study we were unable to source an anti-cathepsin antibody useful for immunostaining of NRK cells, although we were able to support our conclusion of an enzymically-inactive but cathepsin protein-positive population of lysosomes in human MCF7 cells using immunoEM with an antibody to human cathepsin D. For our present study we were still unable to find commercially-available antibodies that gave clear immunostaining of cathepsins in NRK cells. BMV109 reagent does indeed only attach to the active cathepsins[11,12]. Given that our data support the existence of a lysosome regeneration cycle, the covalent attachment of BMV109 occurring in catalytically active endolysosomes does mean that we would expect that eventually the BMV109-conjugated cathepsins will be present in reformed, neutral lysosomes. We have not carried out further experiments to study this.

3) *If not dynasore-sensitive, can the authors suggest alternative mechanisms for tubulation and sorting of the material removed from the sucrosomes?*
Dynasore was expected to block fission not formation of tubules, although as we showed, in the presence of dynasore tubules formed but subsequently collapsed back into the invertase treated sucrosomes (see page 8 of the revised manuscript). We have not yet explored mechanisms of tubulation and sorting into tubules. By analogy with the tubulation necessary for reformation of lysosomes from autolysosomes, possible mechanisms of tubulation include the consequences of production and altered local distribution of phosphoinositides (PIPs), especially PI(3,5)P2[13] and PI(4,5)P2[14] and/or recruitment of the SNX-BAR protein, SNX2 by PI(3,5)P2[15].

Response to Reviewer #2 Issues to be addressed

1- *The V1G1-EGFP and V0a3-EGFP reporters should be validated biochemically for correct incorporation in their respective subdomains. Do they pull down other components of the V1 and V0 sector, respectively, in co-immunoprecipitation assays?*

It has previously been shown that when tagged subunits of the V-ATPase V1 and Vo subcomplexes are immunoprecipitated from transfected cell lysates with anti-tag antibodies there is efficient coprecipitation of other subunits[10]. When we immunoprecipitated either V1G1-EGFP or Voa3-EGFP from stably transfected NRK cells using anti-GFP, we observed co-immunoprecipitation of V1A1 and Vod1, consistent with both EGFP-tagged subunits being incorporated into their subcomplexes and also into complete V-ATPase. The data from a single experiment is shown in Fig C.

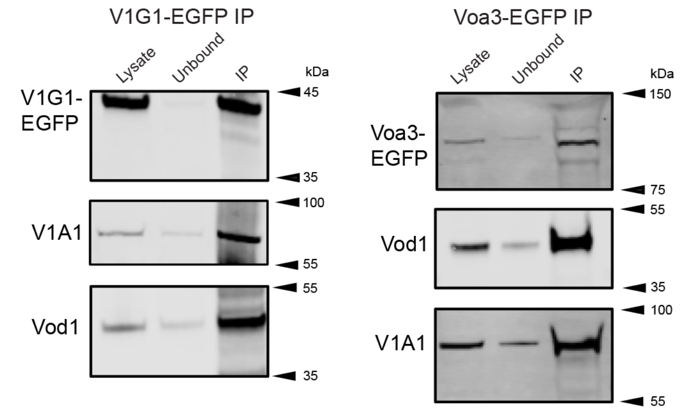

Fig C. Co-Immunoprecipitation of V-ATPase subunits in NRK cells stably expressing V1G1-EGFP or Voa3-EGFP. A. Lysates of NRK cells stably expressing V1G1-EGFP were incubated with an antibody against GFP and Protein A/G magnetic beads to capture V1G1-EGFP and its binding partners. 50μL aliquots, of the original lysate (500μL), the unbound fraction (500μL) and of a 1:10 dilution of the immunoprecipitated (IP) fraction released from the magnetic beads in 50μL SDS-PAGE sample buffer containing 1.33% SDS, were subjected to SDS-PAGE and immunoblotted with antibodies to V1G1, V1A1 and Vod1. **B.** Lysates of NRK cells stably expressing Voa3-EGFP were incubated with an antibody against GFP and Protein A/G magnetic beads to capture Voa3-EGFP and its binding partners. Samples were treated as in panel A and immunoblotted with antibodies to Vod1, V1A1 and GFP for Voa3. In the absence of anti-GFP, none of the V-ATPase subunits were captured on the beads (not shown).

2- *The authors state that autophagy is not triggered by sucrose feeding of NRK cells, but provide no evidence. Given this is likely a major stress, its effect on autophagy should be analyzed. Better still, they could repeat their analysis in NRK cells that are incapable of triggering autophagy due to deletion of an essential regulator (i.e. ATG5).*

We have previously shown by immunofluorescence microscopy that treatment of NRK cells with 30 mM sucrose for 24 hr caused no significant accumulation of LC3-positive autophagosomes (Figure 6E in Bright et al (2016)[9], reproduced as Fig D below) and we reference this finding on page 14 of our revised manuscript. At that time, we also showed no change in amounts of LC3-I and LC3-II by immunoblotting after sucrose treatment (data not shown).

Although we recognise the value of repeating our colocalization analysis of V1G1-EGFP vs LAMP1-mCherry in ATG5-depleted NRK cells, we believe that to do this properly we would need a cell population stably depleted of ATG5 and stably expressing both V1G1-EGFP and LAMP1-mCherry, which we have not yet generated. Although not definitive, we have found no effect of two inhibitors (3μM ULK-101 and 300 nM MRT68921, each for 24h) of the serine/threonine kinase ULK1, the most upstream component of the autophagic machinery[16], on the changes in V1G1-EGFP colocalization with LAMP1-mCherry we observed when sucrosomes were formed and after subsequent incubation with invertase for 8h (data not shown).

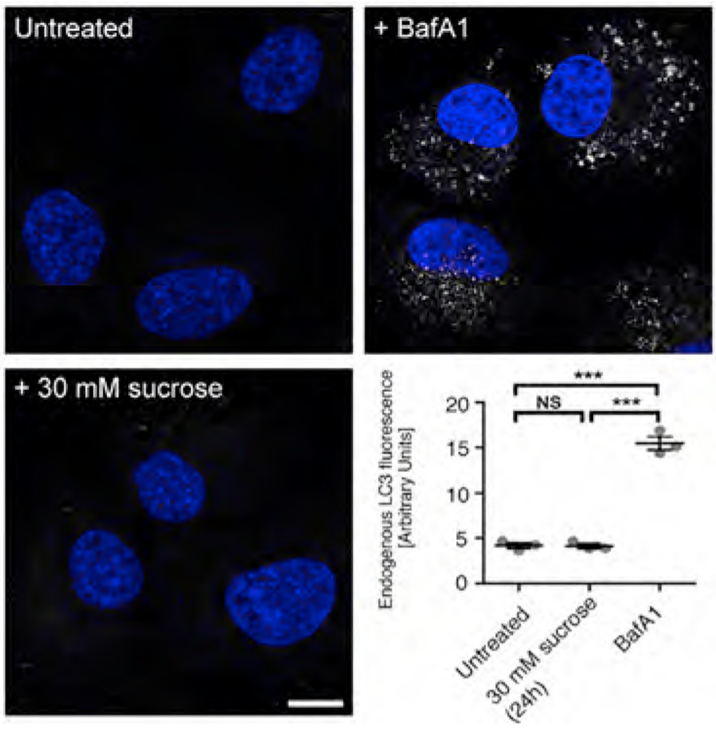

Fig D. Lack of autophagosome accumulation in NRK cells incubated with sucrose. Image is Fig6E from Bright et al (2016)[9]. Anti-LC3 immunoreactivity was used as a marker of autophagosome accumulation in NRK cells following sucrosome formation after incubation with 30mM sucrose for 24h. 400 nM bafilomycin A1 (BafA1) for 8 h was a control for autophagosome accumulation. Nuclei were identified by Hoechst staining and mean LC3 immunoreactivity intensity quantified (mean ±SEM; three experiments), following confocal microscopy. ***p < 0.001 (Student's t test).

3- *Ultimately, the mechanism that couples endolysosomal fusion and content mixing to changes in V-ATPase assembly remains unexplained. One possibility is that the loss of internal acidity due to increased volume (and thus proton dilution) upon fusion may be the stimulus. The authors could test that by measuring V1G1 membrane association and FRAP behavior when cells are treated with agents that disrupt lysosomal acidification, either by directly inhibiting the V-ATPase (i.e. bafilomycin A) or indirectly (i.e. chloroquine).*

Although this is an interesting idea, we think it unlikely that a change in lumenal acidity is the major cause of increased V-ATPase assembly upon fusion of endosomes with lysosomes and we discuss this in the revised manuscript at the bottom of page 14. We have carried out experiments with bafilomycin A as described above in response to point 6 of reviewer#1. In addition, we addressed the FRAP behaviour of V1G1-EGFP associated with sucrosomes in the presence of NH_4Cl. We chose NH_4Cl rather than chloroquine because it had been shown to abolish almost completely the fluorescence recovery of a GFP-tagged V1 subunit in synaptic vesicles[17]. In our experiments fluorescence recovery of V1G1-EGFP was not prevented, but slightly enhanced by the presence of NH_4Cl. Our new data are presented in a new supplemental figure panel (Fig S5I), described on page 11 and discussed on pages 14/15 of the revised manuscript.

References

1. Tuli F and Kane PM (2023) The cytosolic N-terminal domain of V-ATPase a-subunits is a regulatory hub targeted by multiple signals. Front Mol Biosci *10*, 1168680.

2. Chavrier P, Parton RG, Hauri HP, Simons K and Zerial M (1990) Localization of low molecular weight GTP binding proteins to exocytic and endocytic compartments. Cell *62*, 317-329.
3. Bucci C, Thomsen P, Nicoziani P, McCarthy J and van Deurs B (2000) Rab7: a key to lysosome biogenesis. Mol Biol Cell *11*, 467-480.
4. Johnson DE, Ostrowski P, Jaumouille V and Grinstein S (2016). The position of lysosomes within the cell determines their luminal pH. J Cell Biol *212*, 677-692.
5. Humphries WH 4th, Szymanski CJ and Payne CK (2011) Endo-lysosomal vesicles positive for Rab7 and LAMP1 are terminal vesicles for the transport of dextran. PLoS One *6*, e26626.
6. Shearer LJ and Petersen NO (2019) Distribution and co-localization of endosome markers in cells. Heliyon *5*, e02375.
7. Dunn KW, Kamocka MM and McDonald JH (2011) A practical guide to evaluating colocalization in biological microscopy. Am J Physiol Cell Physiol *300*, C723-742.
8. Reaves BJ, Bright NA, Mullock BM and Luzio JP (1996) The effect of wortmannin on localisation of lysosomal type I integral membrane glycoproteins suggests a role for phosphoinositide 3-kinase activity in regulating membrane traffic late in the endocytic pathway. J Cell Sci *109*, 749-772.
9. Bright NA, Davis LJ and Luzio JP (2016) Endolysosomes are the principal intracellular sites of acid hydrolase activity. Curr Biol *26*, 2233-2245.
10. Ratto E, Chowdhury SR, Siefert NS, Schneider M, Wittmann M, Helm D and Palm W (2022). Direct control of lysosomal catabolic activity by mTORC1 through regulation of V-ATPase assembly. Nat Commun *13*, 4848.
11. Withana NP, Garland M, Verdoes M, Ofori LO, Segal E and Bogyo M (2016). Labeling of active proteases in fresh-frozen tissues by topical application of quenched activity-based probes. Nat Protoc *11*, 184-191.
12. Edgington-Mitchell LE, Bogyo M and Verdoes M (2017). Live cell imaging and profiling of cysteine cathepsin activity using a quenched activity-based probe. Methods Mol Biol *1491*, 145–159.
13. Li X, Rydzewski N, Hider A, Zhang X, Yang J, Wang W, Gao Q, Cheng X and Xu H (2016) A molecular mechanism to regulate lysosome motility for lysosome positioning and tubulation. Nat Cell Biol *18*, 404-417.
14. De Leo MG, Staiano L, Vicinanza M, Luciani A, Carissimo A, Mutarelli M, Di Campli A, Polishchuk E, Di Tullio G, Morra V, Levtchenko E, Oltrabella F, Starborg T, Santoro M, Di Bernardo D, Devuyst O, Lowe M, Medina DL, Ballabio A and De Matteis MA (2016) Autophagosome–lysosome fusion triggers a lysosomal response mediated by TLR9 and controlled by OCRL. Nat Cell Biol *18*, 839-850.
15. Rodgers SJ, Jones EI, Arumugam S, Hamila SA, Danne J, Gurung R, Eramo MJ, Nanayakkara R, Ramm G, McGrath MJ and Mitchell CA (2022) Endosome maturation links PI3Kα signaling to lysosome repopulation during basal autophagy. EMBO J *41*, e110398.
16. Zachari M, Longo M and Ganley IG (2020) Aberrant autophagosome formation occurs upon small molecule inhibition of ULK1 kinase activity. Life Sci Alliance *3*, e202000815.
17. Bodzeta A, Kahms M, and Klingauf J (2017). The presynaptic v-ATPase reversibly disassembles and thereby modulates exocytosis but Is not part of the fusion machinery. Cell Rep *20*, 1348-1359.

Revised Supplemental figure legends

Figure S3. Colocalization of EGFP-tagged proteins with LAMP1-mCherry or ARL8b-mCherry in stably expressing NRK cells. (A-E) Pearson's colocalization coefficients for EGFP-tagged proteins vs LAMP1-mCherry in untreated cells, cells incubated with 30mM sucrose for 24h or incubated with sucrose followed by 1-8h incubation with 0.5mg/mL invertase. Mixed stable cells used except in (D) in which a clonal LAMP1-mCherry/ RILP-EGFP cell line depleted of endogenous RILP by siRNA-mediated knockdown (86.7±1.9% (3) depletion) was used. Pearson's colocalization coefficients are shown for exactly the same experiments as the Manders' colocalization coefficients shown in Fig 3 A-E. (**F**) Manders' colocalization coefficients for V1G1-EGFP vs LAMP1-mCherry in mixed stable LAMP1-mCherry/V1G1-EGFP cells otherwise untransfected (control cells) or transiently transfected with 2xHA-RAB7WT, 2xHA-RAB7 Q67L or 2xHA-RAB7 T22N. Transient transfection efficiency estimated by immunofluorescence microscopy as the proportion of cells expressing the 2xHA tag was >75% with all constructs. (**G**) pH of pHLARE-positive organelles in NRK cells stably expressing pHLARE and otherwise untransfected (control cells) or transiently transfected with 2xHA-RAB7WT, 2xHA-RAB7 Q67L or 2xHA-RAB7 T22N. Transient transfection efficiency was >69% with all constructs. Mean pH of pHLARE-positive organelles in 10 cells of 3 separate experiments was calculated as in Fig S1E by comparison with a standard curve derived from pH clamped cells (see Fig S1F). (**H**) Distribution of pH in pHLARE-positive organelles in cells described in panel G. p relative to control in same pH range. **(I, J)** Manders' and Pearson's colocalization coefficients (whole cell, I; outer shell, J) for ARL8b-mCherry vs RAB7a-EGFP in untreated mixed stable ARL8b-mCherry/ RAB7a-EGFP cells, cells incubated with 30mM sucrose for 24h or incubated with sucrose followed by 8h incubation with 0.5mg/mL invertase. The individual data points are for the same cells shown in Fig 3G. **(K, L)** Manders'(K) colocalization coefficient for V1G1-EGFP vs LAMP1-mCherry and Pearson's (L) colocalization coefficient in the presence or absence of 80μM dynasore or 1μM latrunculin A in otherwise untreated LAMP1-mCherry/ V1G1-EGFP mixed stable cells, cells incubated with 30mM sucrose for 24h or incubated with sucrose followed by 8h incubation with 0.5mg/mL invertase. The individual data points are for the same cells shown in Fig 3I. **(M)** Electron micrographs of portions of NRK cells incubated with 30mM sucrose for 24h followed by 2h incubation with 0.5mg/mL invertase ± 80μM dynasore during the second h, showing the

appearance of ‘daisy chains’ of linked swollen sucrosomes after dynasore treatment. In panels A-L, mean ± SEM of 3 experiments (minimum 10 cells per experiment) shown. †, p=0.07; *, p<0.05; **, p<0.01, ***, p<0.005; ****, p<0.001.

Figure S5. The dynamics of V-ATPase V1 subunit recruitment to endolysosomes in live cells. (A-G). Further examples to that shown in Fig 5 A, B of kissing and fusion of individual Dex A546-positive late endosomes with Dex A647-positive terminal endocytic compartments (lysosomes) resulted in the gradual acquisition of Dex A647 (blue lines) in the Dex A546-positive organelle and the rise in recruitment of V1G1-EGFP (green) to the limiting membrane of the resulting endolysosome. Clonal NRK cells expressing V1G1-EGFP were treated and examined exactly as in the Fig5A legend and mean grayscale intensity profiles of Dex A647 (blue) and V1G1-EGFP (green) plotted over time for each Dex A546-positive organelle undergoing kissing and/or fusion that was examined. **(H)** Summary of the time course of increase in V1G1-EGFP fluorescence intensity on the limiting membrane of the organelles shown in panels A-G from the point when Dex A647 begins to increase. **(I)** Alteration in fluorescence intensity in FRAP experiments on sucrosomes in clonal V1G1-EGFP cells (grayscale, mean±SEM). Untreated control, red (n=4); +100nM bafilomycin A1 (1h, 37°C), green (n=8); +50mM NH_4Cl (15 min, 37°C), blue (n=7).

RE: Manuscript #E23-08-0322R
TITLE: "Reversible assembly and disassembly of V-ATPase during the lysosome regeneration cycle"

Dear Paul,

Thank you for resubmitting your manuscript, "Reversible assembly and disassembly of V-ATPase during the lysosome regeneration cycle", to be considered for publication in Molecular Biology of the Cell. Given the positive comments of the reviewers of the original manuscript, I read the revised manuscript and the response to reviewers myself without further reviewer input. In my view, you have more than adequately addressed the original reviewer concerns, and the new data and discussion have strengthened the paper. I find the revised manuscript to be extraordinarily well done, clear, and an important contribution to the field. The data are lovely. Based on this, I am pleased to accept your manuscript for publication in MBoC. Congratulations, and thank you for submitting your work to MBoC!

Sincerely yours,

Mickey
Michael Marks, PhD
Monitoring Editor
Molecular Biology of the Cell

Dear Prof. Luzio:

Congratulations on the acceptance of your manuscript! Thank you for choosing to publish your work in Molecular Biology of the Cell (MBoC).

Within 10 days, an unedited PDF of your manuscript will be published on MBoC in Press, an early release version of the journal. The date your manuscript appears on this site is the official publication date.

Your copyedited and typeset manuscript will be scheduled for publication in the next available issue of MBoC. Our production team will notify you when the page proofs of your paper are ready for your review.

MBoC offers the option to publish your paper with immediate open access. Open access can increase the discoverability and usability of your research. If you would like to publish your paper with immediate open access but did not select this option during initial submission, please contact the MBoC Editorial Office (mbc@ascb.org).

We invite you to submit images related to your accepted manuscript to be considered for the journal cover.
Cover images must contain color, TIF Format
Size image at 7.4" wide x 5.85" deep
RGB and CMYK color (please submit two separate files, one in each format)
300 dpi resolution

Please also send a 1-paragraph description of the image that a general audience could understand. Include with the caption the name and institution of the individual(s) the image should be attributed to. Please provide this description in a Word file. You can send the file via email or by your favorite file transfer service to mboc@ascb.org.

Authors of Articles and Brief Communications are also encouraged to create a short video abstract to accompany their article when it is published. These video abstracts, known as Science Sketches, are up to 2 minutes long and will be published on YouTube and then embedded in the article abstract. Science Sketch Editors on the MBoC Editorial Board will guide you as you prepare your video. Information about how to prepare and submit a video abstract is available at www.molbiolcell.org/science-sketches. Please contact mboc@ascb.org if you are interested in creating a Science Sketch.

We look forward to publishing your paper in MBoC.
Sign up for MBoC's new content alerts here https://tinyurl.com/3bcsyk3e

Sincerely,

Eric Baker

Journal Production Manager
MBoC Editorial Office
mbc@ascb.org
